# Downstream Processing of Therapeutic Peptides by Means of Preparative Liquid Chromatography

**DOI:** 10.3390/molecules26154688

**Published:** 2021-08-03

**Authors:** Chiara De Luca, Giulio Lievore, Desiree Bozza, Alessandro Buratti, Alberto Cavazzini, Antonio Ricci, Marco Macis, Walter Cabri, Simona Felletti, Martina Catani

**Affiliations:** 1 Department of Chemistry, Pharmaceutical and Agricultural Sciences, University of Ferrara, Via L. Borsari 46, 44121 Ferrara, Italy; dlcchr@unife.it (C.D.L.); lvrgli1@unife.it (G.L.); bzzdsr@unife.it (D.B.); alessandro.buratti@unife.it (A.B.); cvz@unife.it (A.C.); 2 Fresenius Kabi iPSUM, Via San Leonardo 23, 45010 Villadose, Italy; antonio.ricci@fresenius-kabi.com (A.R.); marco.macis@fresenius-kabi.com (M.M.); 3 Department of Chemistry “Giacomo Ciamician”, Alma Mater Studiorum—University of Bologna, Via Selmi 2, 40126 Bologna, Italy; walter.cabri@unibo.it

**Keywords:** peptide, biomolecules, biopharmaceuticals, solid phase peptide synthesis, purification, preparative chromatography, continuous chromatography, MCSGP

## Abstract

The market of biomolecules with therapeutic scopes, including peptides, is continuously expanding. The interest towards this class of pharmaceuticals is stimulated by the broad range of bioactivities that peptides can trigger in the human body. The main production methods to obtain peptides are enzymatic hydrolysis, microbial fermentation, recombinant approach and, especially, chemical synthesis. None of these methods, however, produce exclusively the target product. Other species represent impurities that, for safety and pharmaceutical quality reasons, must be removed. The remarkable production volumes of peptide mixtures have generated a strong interest towards the purification procedures, particularly due to their relevant impact on the manufacturing costs. The purification method of choice is mainly preparative liquid chromatography, because of its flexibility, which allows one to choose case-by-case the experimental conditions that most suitably fit that particular purification problem. Different modes of chromatography that can cover almost every separation case are reviewed in this article. Additionally, an outlook to a very recent continuous chromatographic process (namely Multicolumn Countercurrent Solvent Gradient Purification, MCSGP) and future perspectives regarding purification strategies will be considered at the end of this review.

## 1. Introduction

Peptides are organic polymers composed of 2–50 amino acids linked to each other by means of covalent amide (=peptide) bonds. The composition, length and sequence of the amino acid chain have a dramatic influence on the activity of the peptide itself, for example in the human body. Peptides are called bioactive if they have a beneficial impact on body functions, on biological processes and, as a consequence, on health [1,2,3]. The literature about different kinds of bioactivity that peptides can exert on the human body is broad. They can interact with different organs and systems, such as digestive, cardiovascular, nervous and immune systems. This is the reason why they have been extensively studied for their potential applications in cosmetics, food and pharmaceutical fields. Some peptides derived from milk proteins, for instance, are considered promising alternatives to classical treatments for cancer therapy, targeting cancer cells specifically, without affecting healthy ones [1]. Other peptides, as well as some proteins, are considered to be “biomarkers”, which means species that can give an indication about the effectiveness of a treatment [4]. Recently, some peptides have also been tested to treat some symptoms related to COVID-19 disease. For instance, icatibant (a peptidomimetic constituted of 10 amino acids, antagonist of bradykinin B2 receptors and effective against symptoms of hereditary angioedema) has recently shown its potentiality to improve oxygenation in patients affected by COVID-19 at early stage [5,6]. Moreover, some peptidic vaccines have also been designed to provide immunity to SARS-CoV-2 [7].

This class of molecules represents an effective alternative to small molecule drugs. Peptides, especially if endogenous, exhibit remarkable advantages such as high bioactivity, specificity to the target tissues, broad range of therapeutic effects, no toxicity nor tendency to accumulate in the human body [8,9,10,11,12,13]. For instance, classical drugs with anticancer or anti-carcinogenic activity usually cannot recognize cancer cells from normal ones, and this causes an intrinsic toxicity of “traditional” therapies. Peptide-based treatments not showing this negative effect are being considered more and more appealing [1].

In 2014, about 60 peptide drugs had already been approved and launched on the market. At that time, the peptide market represented about 1.5% of all pharmaceuticals sales [14]. The increasing number of peptides entering clinical trials year by year indicates that there is a growing interest from the side of pharmaceutical companies in the use of this class of molecules as drugs [14,15,16]. To date, the number of therapeutic peptides approved is 70 [17].

Peptides can be classified as endogenous, if they are synthesized inside the human body, or exogenous if they are introduced into the organism from an external source. For instance, it is well-known that proteins acquired through food are an essential source of amino acids and it has been demonstrated that some specific portions of dietary proteins exert biological functionalities. Very important sources of bioactive peptides include dairy food, fish, eggs, soybean, rice, corn, peanuts etc. In most cases, the amino acid sequence of the bioactive peptide is contained within a parent protein, where it is inactive, and it can be released by enzymes during digestion [18,19,20]. In other cases, the peptide of interest can be synthesized. Recently, peptidic analogues possessing remarkable pharmaceutical properties have been developed and obtained by means of chemical synthesis [21].

The most suitable technology for the production of a given peptide strongly depends on the molecular size of the target molecule [22]. Even though often highly selective, all production methods lead to mixtures where the target peptide is present together with a series of other molecules (impurities). The target Active Pharmaceutical Ingredient (API) must be separated from all impurities produced during the manufacturing process, because strict regulations are imposed to all kinds of pharmaceuticals, for quality and safety reasons. Particularly, impurities can be divided into two groups [23,24]:

- Process-related impurities, deriving from the production method employed (salts, pieces of cells or of DNA, …)
- Product-related impurities, that are species chemically similar to the target product.

The first type of impurities can be easily separated from the peptide of interest, e.g., through affinity chromatography, during the so-called capture step [25]. In this chromatographic technique, the ligand specifically binds to the target molecule, whereas the impurities are flushed through the column. The peptide or protein is then eluted with a suitable buffer.

Elimination of product-related impurities (which is technically defined as the polishing step of the purification process) is much more challenging because of their similarity to the target molecule, that makes the use of affinity chromatography impracticable [24]. In the vast majority of cases, even this step is performed by means of liquid chromatography.

It is worth mentioning that the capture step is only performed for purification of peptides manufactured with recombinant methodologies, therefore capture will not be discussed any further; on the other hand, the different solutions proposed to perform the polishing of complex mixtures will be subject of this review. The paper will conclude with an overview on the future perspectives in the field of chromatography for the purification of peptides and other biomolecules, including the emerging continuous chromatographic processes.

## 2. Methods of Production (Upstream Processing)

Production of the peptide occurs during the upstream part of the manufacturing. There are several ways to obtain the target peptide. Some of them imply to extract it from the parent protein, where the peptide is contained but inactive. The release is performed through the action of enzymes or by microbial fermentation. Other processes involve to synthesize the peptide-chain starting from single amino acids, adding one amino acid at a time [18].

### 2.1. Enzimatic Hydrolysis and Microbial Fermentation

Food is a valuable source of amino acids and peptides. For example, proteins contained in food can release peptides with bioactive functions during their fermentation or when exposed to enzymes with hydrolytic activity. The outcome of the hydrolysis, namely the type of peptides produced starting from a single parent protein, depends on many experimental factors (type of enzyme or microorganism used, combination of vitro enzymatic hydrolysis with microbial fermentation, etc.). Therefore, the number of bioactive peptides obtainable from food proteins is essentially unlimited [3]. Dairy products, for instance, are an important source of proteins in the human diet and, from them, a number of bioactive peptides can be obtained. Peptides with antihypertensive, antibacterial and immunomodulatory activity have been released by casein and by whey proteins using pepsin, trypsin and chymotripsin as enzymes [26,27,28,29]. Additionally, the enzyme thermolysin has been employed to obtain hypotensive peptides from other kind of foods, such as corn and porcine skeletal muscle [30,31,32,33]. The traditional mode to perform hydrolysis of proteins is to operate in batch, which means through a discontinuous process inside a reactor. This method, anyway, has resulted to be less efficient than continuous methods employing enzymatic membrane reactors, where protein hydrolysis, product collection and catalyst recovery happen in the same unit [34,35].

Dairy products can release bioactive peptides also when subjected to the action of particular bacteria that trigger the fermentation of this kind of foods. For example, it has been demonstrated that *Lactobacillus helveticus*, *Enterococcus faecalis*, yoghurt and cheese bacteria and Lactic Acid bacteria can hydrolyze milk proteins to produce peptides with ACE-inhibitory activity (antihypertensive) [28,36,37,38,39,40,41]. Similarly, peptide with this kind of bioactivity were produced from chicken meat, using *Aspergillus* protease [42].

Another kind of bioactivity that was observed in protein hydrolysates deriving from beef meat is the antioxidant one, especially against lipid oxidation. The employment of bioactive peptides as antioxidant additives in food could be pivotal in the substitution of artificial antioxidants, whose potential health risks are already recognized [43,44].

### 2.2. Chemical Synthesis

Despite the success of other production methods, the technique of choice to produce small to medium peptides, especially for pharmaceutical applications, is still chemical synthesis [45]. The main reasons are two: firstly, a synthesis method is developed starting from standard and well-established procedures, so its development is less complex and less time-consuming. Secondly, in the synthetic approach, differently than recombinant approach (see Section 2.3), modified amino acids can also be incorporated in the peptide chain. These characteristics make chemical synthesis the preferred technique for peptide production.

**Figure 1:**
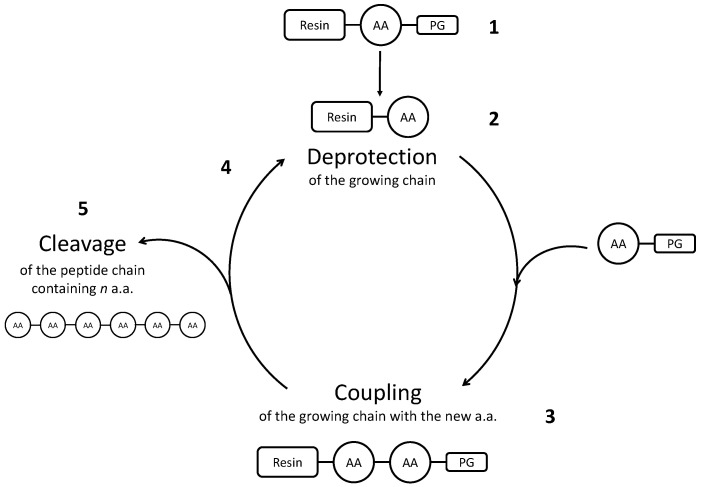
Scheme representing Solid Phase Peptide Synthesis. (1) An amino acid (AA) is protected on the functional group that must not react, and it is bonded to an insoluble resin. Then, its protecting group (=PG) is removed (2), so that the following amino acid, which in turn is protected, can bond to the growing chain (3). Successively, also the second amino acid is deprotected, to add the third one (4). After all the amino acids have been added, the peptide chain is recovered from the synthesis mixture with the step called cleavage (5).

Chemical synthesis can be performed either in liquid- or in solid-phase. Both strategies are based on similar reaction mechanisms, where amino acids and/or fragments of the desired peptide are added successively to the mixture, to react with the growing chain. In Solid Phase Peptide Synthesis (SPPS), firstly developed by Merrifield [46], one N-protected amino acid reacts with the peptide chain, which is anchored to a solid support (a resin) and, after that, the terminal amino acid is deprotected. Then, the following amino acid undergoes the same procedure. Functionalities on the aminoacid side chain need to be protected as well, in order to avoid side reactions. After the procedure is terminated and all the amino acids have reacted, the peptide is released from the resin during the cleavage step. A scheme representing the Solid Phase Peptide Synthesis is depicted in Figure 1.

The presence of the solid support allows one to recover the product simply by filtration: in this lies the reason of the success of synthesis in solid phase. Moreover, the process can be automated [14,22,47,48,49]. Currently, huge efforts are being made in order to make the synthetic processes as green as possible, by introducing the use of protecting groups and alternative solvents more sustainable than the traditional ones [50,51,52].

Liquid phase synthesis plays an important role in the manufacturing of short peptides (up to 10 amino acids). Recently, this approach gained importance for the manufacturing of longer peptides through the coupling of its previously synthesized fragments [53].

### 2.3. Recombinant Approach

This technique is the preferred one to produce peptides containing only natural amino acids on large scale. Compared to isolation from proteins and chemical synthesis, recombinant approach represents the most cost-effective and green way for large-scale peptide manufacturing. Particularly, *Escherichia coli* is the most widely used host. With this genetic engineering process, nevertheless, only peptides containing natural amino acids can be produced. Moreover, the biotechnological process requires great research efforts to develop a suitable procedure, and it is also time-consuming. Generally, the steps followed are: selection of an appropriate expression system, construction of expression vectors, development of the bioprocess. The operating conditions can be tailored for the specific product considered [14,47,54].

## 3. Purification Techniques (Downstream Processing)

None of the aforementioned upstream methods to obtain the peptide of interest leads to a single product. Actually, a series of impurities are produced together with the target. They must be removed during the downstream step of manufacturing. The reason is that every impurity could potentially exert adverse biological activity on the human body. Therefore, very strict purity requirements are applied to pharmaceuticals.

Different purification methods have been developed, with their own advantages and disadvantages. The purification strategy must be evaluated for every single case, in light of imposed requirements that, in turn, vary depending on the particular application [55].

If a peptide is produced through hydrolysis, for instance, it can be separated by means of ultrafiltration from the enzyme employed during the process and from other protein residues with higher molecular mass. Generally, in this case, the membranes of choice have a low molecular mass cut-off and the size of their pores depends on the molecular weight of the desired peptide. Anyway, the main disadvantage of this technique is the poor selectivity of the membrane [1,56].

A technique particularly useful in case of separation of charged peptides and proteins is IsoElectroFocusing (IEF), which is based on the same separation principles as electrophoresis. The sample is injected in a chamber where an electric field is applied, in presence of a pH gradient. Acidic species move towards the anode and basic ones towards the cathode. When a species reaches a zone with pH identical to its isoelectric point, it stops migrating. Then it can be moved to a detection windows to be identified. Therefore, IEF separates analytes depending on their isoelectric point [57]. Several modes of IEF have been developed, some of which can be used on analytical while others on preparative scale, which is the case of IEF in solution [58] or in a cellulose-based separation medium [59]. Anyway, IEF lies outside the main topic of this review and therefore it will not be treated any further.

A common purification issue is the separation of a complex mixture of peptides deriving from solid-phase synthesis. The separation can only be done after cleavage and it is frequently challenging because impurities may differ from the target peptide by a single amino acid or a single functional group, resulting in very similar chemical properties [60]. Typical by-products due to solid phase synthesis include peptides with one amino acid that did not react or reacted in the wrong position, peptides with side-chain modification (oxidation, deamidation, epimerization, alkylation, ring closure or opening, incomplete deprotection) and truncated peptides [61,62].

Chromatography is the most suitable technique for the purification of valuable products, which is the case of pharmaceuticals, where high resolution and selectivity are required [63,64]. This technique allows one to obtain very high efficiency in the separation of complex mixtures, where the components have similar chemical properties. It is flexible and adaptable since a wide selection of stationary and mobile phases to choose from is available. Additionally, a number of well established chromatographic methods have already been developed at industrial level and are available for practical biopharmaceutical applications [55]. A disadvantage of chromatographic methods is that it is difficult to handle viscous mixtures, which cause increase in the backpressure; moreover, organic solvents are used almost always as mobile phase, and this poses environmental concerns regarding their toxicity and disposal [65]. Anyway, chromatography remains the technique of choice for the purification of biomolecules at laboratory, preparative and industrial level. Currently, industrial processes for biopharmaceuticals employ almost exclusively chromatography both for capture and for polishing steps, whose difference has already been explained in Section 1.

Due to the complexity of the peptide mixtures, generally a combination of chromatographic techniques based on different separation principles is required to improve the resolution power [66]. Therefore, at least two different chromatographic modes are applied consecutively, either online or offline, resulting in a multidimensional separation (e.g., two-dimensional liquid chromatography, 2D-LC). A separation performed on the basis of different types of interactions is often referred to with the term “orthogonality” [67], meaning that the two dimensions of the separation can remove different impurities. For example, ion-exchange, HILIC and reversed-phase chromatography separate the analytes depending on different features (charge, hydrophilicity and hydrophobicity) and therefore they can be used as dimensions orthogonal to each other [68]. Otherwise, also reversed-phase in acidic conditions and in basic conditions can be considered to be orthogonal separation methods and have shown very good results in terms of peak capacity when applied to purifications of peptide mixtures [69]. In the case where the two chromatographic modes are coupled offline, the product eluting from the first column is collected and then re-analyzed in the second dimension. This approach is quite labor-intensive and time-consuming. On the other side, in online multidimensional chromatography the product eluting from the first column is immediately injected into the second column, and this allows one to speed up a lot the analysis time, but requires compatibility between the solvents used in the two dimensions. For preparative scale purification, the heart-cutting mode is generally the preferred choice, meaning that only the peak of interest is further separated in the second dimension [70]. On the contrary, for analytical scale analysis, meaning to identify components in the mixture, comprehensive multidimensional separations are usually performed, where the eluent from the first dimension is injected into the second column over the entire first separation time [71]. Generally, mass spectrometry is coupled to multidimensional chromatography, especially at the outlet of the second column [72].

Multidimensional chromatography is based on the same principles as one-dimensional chromatography and therefore will not be further discussed. In the next paragraphs, different modes of chromatography will be considered in detail. Additionally, different purification techniques described in this Section and in the next ones are summarized in Table 1.

### 3.1. Reversed-Phase Liquid Chromatography (RP-LC)

The mode of chromatography most frequently encountered when it comes to the purification of peptide and protein mixtures is reversed-phase liquid chromatography [73]. This technique separates the analytes depending on their hydrophobic properties [1]. Generally, C18 ligands are the most employed stationary phases in RP-LC, but occasionally C8 or even C4 ligands have shown better retentive characteristics, especially in case of very hydrophobic peptides. In other cases, also monolithic, poly(styrene-divinylbenzene)-based columns have been used [74]. All these stationary phases are able to distinguish diastereomers (peptide epimers for instance) but not enantiomers.

In RP-LC, the retention of macromolecules, such as peptides, decreases drastically with the content of organic modifier [48,75,76]. Therefore, it is recommended for macromolecules to use gradient elution, which also contributes to improve the separation of the target peptide from its product-related impurities, since species with similar structure can show very different adsorption behaviour at a given mobile phase composition [49]. Several peptide mixtures have been separated by means of gradient elution in RP-LC, such as insulin from its main degradation product (A21-desamido insulin) [77] and octreotide from impurities [49]. Using a very shallow gradient (0.1% ACN per min), Harris and coworkers [78] managed to purify mixtures of different synthetic polypeptides, with length ranging from 23 to 51 amino acids, containing closely eluting impurities. Sample amounts varying from 145 to 900 mg of peptide mixture could be purified with this one-step chromatographic method, including peptides modified with non-proteinogenic substituents (e.g., biotin, carboxyfluorescein); the purities reached were almost always above 95%.

Besides ACN and other more eco-friendly solvents have been tested and compared to it. Ethanol, for instance, has shown elution strength and separation characteristics similar to ACN during the separation of three peptides (bradykinin, angiotensin II, angiotensin I), thus resulting to be a promising candidate to substitute ACN in some cases [79].

Acidic ion-pairing agents (trifluoroacetic acid or formic acid) are often added to the mobile phase to pair with basic amino acids, that are positively charged at acidic pH, improving peak shape [4,80]. A study demonstrated that TFA concentration affects the recovery of the peptide or protein: at low TFA concentrations, below 0.05%, strong ion-exchange interactions can establish between analytes and hydrolyzed silanols, thus causing peak broadening [81].

Mazzoccanti et al. [82] developed a d-ERRP (dynamic Electrostatic-Repulsion Reversed-Phase) method (a variation of the classical static ERRP) based on the repulsion between the basic peptide and a hydrophobic ion-pairing agent adsorbed on the alkyl chains of the stationary phase, both positively charged at acidic pH. The hydrophobic agent used in that research was tetrabutylammonium, dissolved in the mobile phase together with TFA. This innovative chromatographic mode was successfully applied to the purification of glucagon (containing 29 amino acids) from its epimer [D-His]^1^-Glu and other four critical synthetic impurities deriving from deamidation or racemization of some amino acids. This technique is called “dynamic” because the repulsion is generated by the flow of the mobile phase, in opposition to static ERRP that will be discussed later.

A remarkable advantage of RP-LC over other chromatographic modes is that, thanks to the solvents employed, it can be easily coupled with Mass Spectrometry, a detection technique very popular for the characterization of macromolecules such as peptides and proteins [83]. This technique has allowed one, for instance, to separate bioactive peptides and tryptic digests of different proteins in RP-LC under both acidic and alkaline conditions, using trifluoroacetic acid or a buffer made of triethylamine and acetic acid respectively, and applying a gradient of ACN [74]. The detectability of peptides in conditions of full-scan negative-ion electrospray ionization mass spectrometry after the separation at high pH was two to three times lower with respect to their detectability in conditions of full-scan positive-ion electrospray ionization mass spectrometry after the separation at low pH.

### 3.2. Ion-Exchange Chromatography (IEX)

In ion-exchange chromatography, the separation mechanism is based on the electrostatic interactions between the opposite charges of analytes and stationary phase. Separation is modulated by the amount of competitive ions present in the mobile phase [1]. This technique is particularly suitable when dealing with peptide purification, because most peptides have a net charge that can be varied depending on the pH of the mobile phase. At acidic pH, carboxylate groups and basic residues (Arg, Hys, Lys) are mainly protonated. Therefore, IEX is often used to characterize charge variants of peptides and proteins, even though lately it has been more and more replaced by RP-LC because of its incompatibility with MS detection [84].

To identify peptide modifications such as deamidation or acetylation, that are detected with difficulty through RP-LC, IEX reveals to be a good choice. Moreover, this technique is able to distinguish between analytes with similar hydrophobicity. For example, using a strong cation-exchange column (Luna SCX containing a phenyl sulfonic acid exchanger) with salt gradient (namely, potassium chloride) it was possible to separate bradykinin variants differing only slightly in hydrophobicity or only by one charge [85].

**Figure 2:**
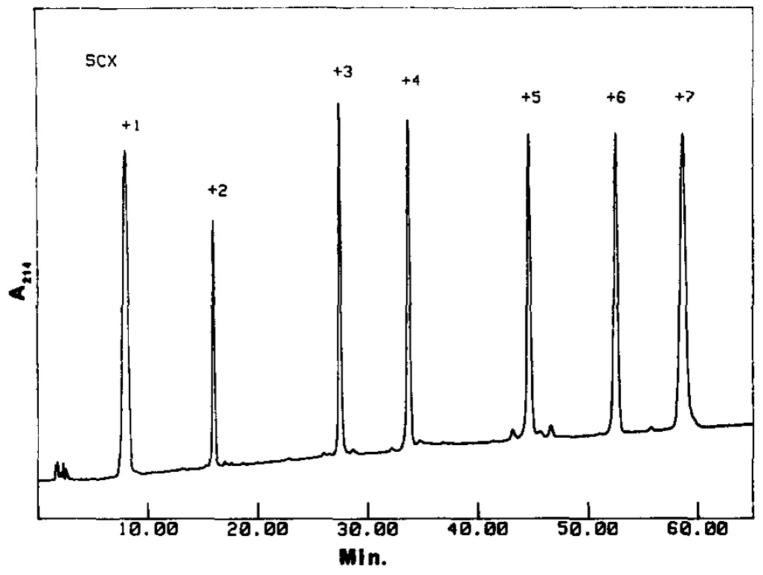
Separation of seven synthetic peptides bearing different charges by means of Ion-Exchange chromatography. Reproduced with permission from [86].

Crimmins [86] verified that, during the IEX separation of a complex mixture of synthetic peptides positively charged from +1 to +7 at pH 3, the order of elution followed the order of charge monotonically; the most retained compound was the peptide with charge +7 (see Figure 2). In that case, the stationary phase used was a sulfoethyl aspartamide (a hydrophilic strong-cation exchange adsorbent), whereas mobile phases were MP_*A*_ = 5 mM sodium phosphate pH 3, 25% acetonitrile (ACN), and MP_*B*_ = 5 mM sodium phosphate, 500 mM NaCl pH 3, 25% acetonitrile. At low levels of ACN, retention and selectivity are mainly governed by the presence of basic amino acids and positive charges contained in the peptide. The same stationary phase has been tested by different research groups to analyze several peptides (up to 50 in a single study, ranging from 5 to 20 amino acid residues) [87,88,89]. However, the elution order does not always follow the order of charge. By analysing with the same method described above the peptide fragments produced by digestion of myoglobin, it was noted an inversion of the elution order with respect to the charge order [90]. This behaviour was attributed to the overall hydrophobicity of the peptide and to the fact that, for steric reasons, the charged residues do not interact simultaneously with the ion-exchange resin. Probably, there is a limit related to peptide molecular weight above which the monotonic relation between the elution order and the global charge of the analyte is no longer valid.

Ion-exchange chromatography has been employed also for pre-purification of the peptide of interest. In a previous study, lactoferrin was hydrolyzed with porcin pepsin A. The hydrolysate was then loaded in a SP-Sepharose Fast Flow column (where SP is sulphopropyl, a strong cation exchanger) and eluted with a gradient of ammonia solution. During the gradient elution, only impurities were eluted. At the end of the gradient, a wash was performed with NaCl 2 M, in order to recover the target peptide (LFcin-B) [91]. Otherwise, it is possible to modulate the experimental conditions in order to trap the impurities on the resin whereas the peptide passes through the column with no retention. For example, this procedure was applied for the purification of C-peptide. This molecule contains 31 amino acid residues, with only one being basic, therefore it has a very low isoelectric point, around 3. By using a strong cation exchange and applying a mobile phase with pH slightly above its isolectric point, this strategy resulted to be successful, as it was confirmed by a comparison of LC-MS chromatograms of the sample before and after the purification [92].

Last, IEX is often employed for peptide mapping, to demonstrate protein identity, as in the case of cytochrome C tryptic digest and hemoglobin [84].

### 3.3. Hydrophilic Interaction Chromatography (HILIC)

**Figure 3:**
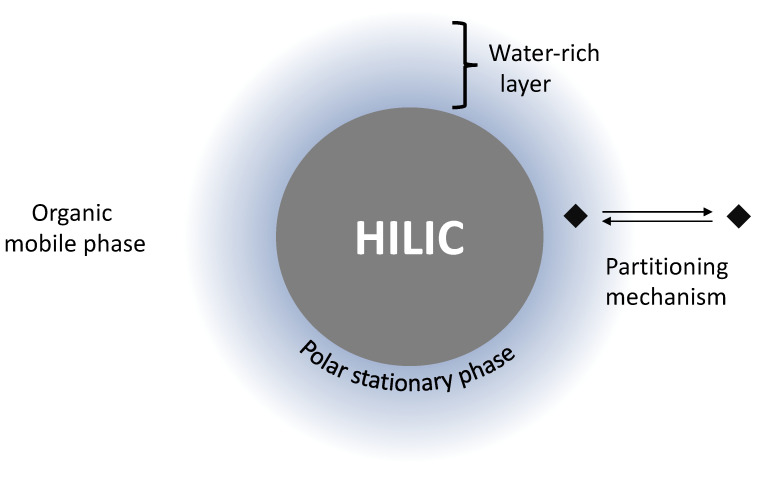
Scheme of the mechanism regulating elution in HILIC. The polar stationary phase is surrounded by a layer rich in water, whereas the mobile phase is an organic solvent. Probably, the analytes are retained because of partitioning mechanism. It results that more hydrophilic components are more retained.

Hydrophilic Interaction Chromatography (HILIC) and can be considered as a variant of normal-phase chromatography. As it is shown in Figure 3, it is likely that HILIC involves a partitioning mechanism between the more hydrophobic mobile phase and a layer of solvent rich in water adsorbed on the stationary phase, but the phenomena responsible for such a behaviour are still not well understood [93]. Alpert [87] demonstrated that, if at low percentage of ACN the separation mechanism is mainly based on ion-exchange, on the other hand retention increases dramatically at high levels of ACN (e.g., greater than 50%). This is due to hydrophilic interactions. At the same time, electrostatic effects diminish in importance. The order of elution is the opposite than in reversed-phase chromatography, meaning that retention increases from least to most hydrophilic component [94].

HILIC has been employed for different applications. Nine peptides present in an illicit drug mixture, including oxytocin, leuprorelin, sermorelin, epitalon and melanotan II, have been separated within 35 min using a gradient elution method employing LC-DAD-MS. The mobile phases used for the gradient were MP-A: acetonitrile, deionized water and 100 mM ammonium formate pH 3.0 (80:10:10, *v*/*v*/*v*), and MP-B: acetonitrile/deionized water/100 mM ammonium formate pH 3.0 (40:50:10, *v*/*v*/*v*). The column was a ZIC HILIC column, made of bare silica modified with sulfobetaine. In addition, other stationary phases were tested, including CORTECS (made of bare silica) and BEH HILIC column (composed of unbound porous particles hybridized via ethylene crosslinking) [95]. A comparative study between three HILIC columns (XBridge amide from Waters, bare silica Kinetex HILIC from Phenomenex and silica with quaternary ammonium and sulphonic acid Nucleodur HILIC from Macherey Nagel) was accomplished in the context of quantification of proteotypic tryptic peptides, where chromatography was coupled with ESI-MS. It resulted that a higher sensitivity was obtained using the amide column without salt buffer in the mobile phases [96]. HILIC technique proved to be a good solution also for the separation of immunoglobulin deriving peptides containing modified residues (deamidated asparagine and oxidized methionine) from their native forms, a procedure necessary for the identification and quantification of critical impurities. The method involved the use of a Penta-HILIC column and a gradient elution program where the percentage of strong solvent (water containing 0.1% formic acid and 50 mM ammonium formate) increased from 5 to 70% within 90 min, whereas the content of ACN decreased [97].

**Figure 4:**
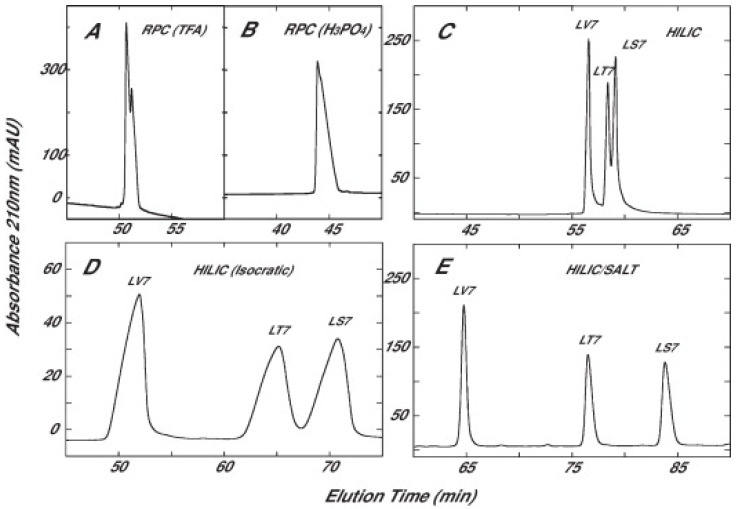
Separation of a mixture of LV7, LT7 and LS7 peptides by RP-LC with TFA (panel **A**) or phosphoric acid (panel **B**) as the ion-pairing reagent, HILIC (decreasing concentration of acetonitrile, panel **C**), isocratic HILIC (isocratic concentration of acetonitrile, panel **D**) and HILIC/SALT (isocratic concentration of acetonitrile and salt gradient, panel **E**). Reproduced with permission from [98].

A variation of the classical HILIC technique, developed by Mant et al. envisaged to perform the peptide separation using a salt gradient (sodium perchlorate) in the presence of isocratic high content of organic solvent, specifically ACN around 80–90%. The column employed was a Halo Penta-HILIC column. This technique was applied to different mixtures of synthetic peptides differing in structure, number of amino acids (from 10 to 26) or charge, and in those circumstances it resulted to be more suitable for peptide separations than the traditional version of HILIC in terms of resolving capabilities [98]. For instance, Figure 4 shows the differences in the separation of a mixture of three synthetic *α*-helical peptides by using reversed-phase chromatography (panels A and B), HILIC (panel C), isocratic HILIC (panel D) and HILIC/SALT (panel E). The peptides considered have the same structure (Ac-ELEKLL**X**ELEKLLKELEK-amide) except for the amino acid **X** in position 7, which in the three cases is either Ser (LS7), Thr (LT7) or Val (LV7). Ser and Thr are much more polar than Val, which is non-polar, and this explains the order of elution.

### 3.4. Mixed-Mode Chromatography

**Figure 5:**
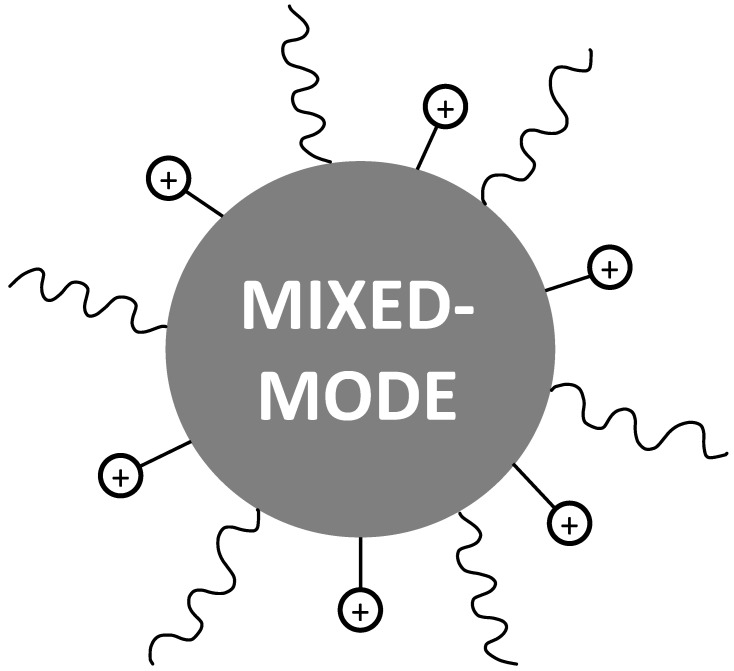
Scheme of a typical stationary phase used for Mixed-Mode Chromatography. The particle is functionalized with alkylic chains (e.g., C8) and charged groups (e.g., quaternary amines).

Peptide mixtures contain a huge number of impurities with different chemical properties. Therefore, a single chromatographic technique is often insufficient to obtain a good separation. Both hydrophobicity/hydrophilicity and charge play a key role in the purification of peptide samples. Recently, innovative stationary phases have been developed, that combine two separation mechanisms (reversed-phase or HILIC and ion-exchange) [99,100]. The presence of two different ligands enables two separation mechanisms: a first one based on the hydrophobic or hydrophilic character of the compound of interest and the second one based on its ionic properties, namely its net charge at the pH of the mobile phase [60,101]. An example of stationary phase containing both hydrophobic chains and positively charged ionic groups is shown in Figure 5.

Moreover, depending on the ion-exchanger nature, on the pH of the mobile phase and on the pI of the peptide of interest, the ion-exchange mechanism can work either in attractive or in repulsive way. In 2014, Gritti and Guiochon [102] applied the static ERRP concept by using BEH-C18 columns modified with different loadings of quaternary amino groups. Since the positive charges are chemically bonded to the silica surface, this ERRP can be defined “static” in opposition to dynamic ERRP, described in Section 3.1. They applied this technique to several peptides and proteins, with molecular mass ranging from 0.9 to 80 kDa and pIs between 4.7 and 11.3. Water and ACN with TFA were used as mobile phases (pH = 3); being the pH of the eluent lower than all the PIs of the analytes, it resulted that all the peptides and proteins as well as the amines were positively charged, and therefore the ion-exchange mechanism worked in repulsive way. As expected, the retention times of all compounds decreased with increasing surface density of the amino groups on the stationary phase, because of the greater electrostatic repulsion between the positively charged analytes and the ionic groups bonded on the BEH-C18 stationary phase.

Khalaf and coworkers [100] used Zeochem columns functionalized with C8 chains and with quaternary amines. The mobile phases tested were aqueous buffers containing different salts (sodium acetate, ammonium acetate) and a percentage of ACN between 3 and 50% v/v. When pH is lower than pI both the ion-exchangers and the peptide are positively charged; therefore, their interaction is repulsive, and, as a consequence, the global effect is to decrease the retention times. In that research, Khalaf demonstrated that significant improvements in separation performance were obtained, both in analytical and in preparative scale: selectivity and productivity were increased up to twice as high, whereas yield was improved by around 20%.

Kadlecová et al. [103] compared two columns with positively charged ionic ligands (XSelect CSH C18, containing pyridyl group, and Atlantis PREMIER BEH C18 AX, containing a quaternary alkylamine) for the separation of 14 peptides, especially ten dipeptides, three pentapeptides and one octapeptide. The analytes have been separated in gradient conditions using ammonium formate 5 mM (pH 3) and ACN. All the peptides were baseline separated within four minutes using PREMIER BEH C18 AX column and a quite steep gradient. To obtain a comparable degree of separation, a more shallow gradient had to be used on XSelect CSH C18, achieving a complete separation in 10 min.

On the other hand, for the purification of a goserelin mixture, Bernardi et al. [99] used Zeochem columns functionalized with C8 chains and sulfonic groups (strong cation-exchange ligands). The mobile phases were aqueous buffers containing sodium acetate in different percentages and a known quantity of ACN (from 6 to 50% *v*/*v*). pH was around 4, while pI of goserelin was 11.5: therefore, the electrostatic mechanism was attractive, because the analytes and the ionic ligands on the stationary phase bore different charges. They demonstrated that the adsorption strength increases with increasing the percentage of sulfonic groups on the surface of particles: higher Henry coefficient values were found for higher ion-exchanger concentrations.

Beside hydrophobic compounds, also hydrophilic analytes can be separated in mixed-mode. Litowski et al. [60] used a polysulfoethyl A strong cation-exchange column, that presents a hydrophilic character, to separate in gradient conditions a mixture of a 21-residues synthetic amphipathic *α*-helical peptide from its impurities modified on the side-chain, particularly acetylated on three different serine residues (positions 3, 10 and 17). Eluent A was 10 mM aqueous TEAP, pH 6.5, containing 65% ACN and eluent B was identical to eluent A but contained also 350 mM NaClO_4_. The ability of HILIC/CEX of separating these four species is outstanding, since the analytes exhibit the same charge and extremely similar hydrophilicity, features that require both separation mechanisms to isolate the target compound.

## 4. Current Challenges and Future Perspectives

### 4.1. The Need to Investigate the Theoretical Basis of Adsorption

The goal of purification procedures is to achieve high purity products with elevated recovery, possibly by means of high throughput and economically viable processes. Combining all these requirements is challenging. Moreover, during the development of purification processes, often a “trial-and-error” strategy is followed, an approach which is time-consuming and costly. Indeed, it is estimated that around 50 to 70% of the whole manufacturing cost is attributable to the downstream processing [49,65,104]. In this regard, it appears necessary to model the chromatographic behavior of the species of interest through theoretical and computational instruments, in order to define a priori proper experimental conditions for the purification of the target [105,106]. Particularly, knowing the thermodynamic properties (that is, the adsorption isotherms) involved in the adsorption of the target molecule is the basis to predict its behaviour in a wider range of working conditions, which makes trial-and-error optimization superfluous. Additionally, mechanistic models can be a useful tool to predict the impact of process parameters and experimental conditions on product quality [107]. Computational methods, such as Inverse Method, allow one to obtain thermodynamic data starting from very little amount of compound, which is particularly advantageous when the material of interest is expensive or present in limited amount, and this could promote the need for model building among pharmaceutical companies during process development phases. Some of the authors of this review have published a study regarding the modeling of the chromatographic behaviour of a peptide in non-linear conditions [49].

The investigation of thermodynamic adsorption equilibria regulating the separation in batch of the compounds contained in the peptide mixture could also be used to scale the process to continuous chromatography, a field where the knowledge of non-linear chromatography has not been deeply investigated yet.

### 4.2. Continuous Chromatographic Techniques

**Figure 6:**
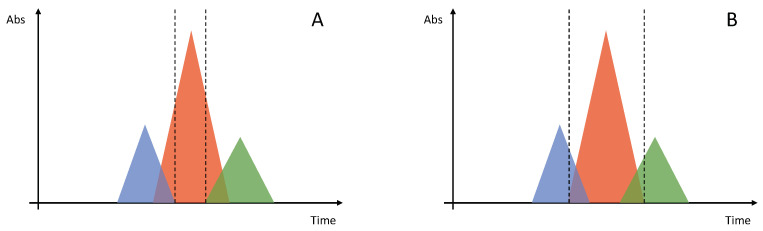
The red peak represents the target product, eluting between a group of impurities more weakly adsorbed (blue peak) and a group of impurities more strongly adsorbed (green peak). When deciding which portion of the main peak to collect, two cases are possible: (**A**) narrow collection window leads to higher purity but lower recovery; (**B**) broad collection window leads to high recovery but low purity, since also a portion of impurities peaks is collected. This is the so-called “purity-yield trade-off”.

The chromatographic processes performed during the polishing steps usually employ a single column; this operative methodology is called “batch”. The classic situation encountered in these purification processes is a ternary separation, meaning that the peptide of interest elutes as intermediate between two groups of impurities: a first group with lower retention (more weakly adsorbed on the stationary phase) and a second group with higher retention (more strongly adsorbed). The similarity between the target product and its related impurities leads unavoidably to an overlap between their peaks, on both the front and the tail of the product peak; the situation becomes even worse for higher loadings. As a consequence, two borderline cases are possible: the collection window can be narrowed, in order to obtain a higher purity, to the detriment of recovery, or it can be widened, and this leads to higher yields but lower purity, since also a part of the impurities peaks is collected. The qualitative example depicted in Figure 6, shows that in batch chromatography it is practically impossible to reach both a high purity and high yield at the same time (yield-purity trade-off) [108,109].

Multicolumn chromatographic processes can help to overcome this limitation, very frequent for complex mixtures of biopharmaceuticals. This group of techniques includes processes where at least two columns, identical in dimensions and in stationary phase, are used. The concept on which multicolumn processes are based is the countercurrent movement of the mobile phase with respect to the stationary phase, simulated through a series of switching valves, that can influence the path accessible to the mobile phase. In most cases, the countercurrent movement of the two phases allows to improve the recovery. The second fundamental aspect of multicolumn processes is that they work continuously, making the whole procedure cyclic and automatic. This permits to reduce times related to operations that the operator would accomplish manually and, as a natural consequence, it allows to improve reproducibility.

Particularly, to deal with polishing of complex biomolecules mixtures, a process called Multicolumn Countercurrent Solvent Gradient Purification (MCSGP) has been developed around fifteen years ago by Aumann and Morbidelli [110]. During the years, this appealing technology has found a wide application range, since it has been used for several cases of biomolecules purification where intensification of the downstream steps was needed, such as the purification of monoclonal antibodies [111,112,113], PEGylated proteins [114], oligonucleotides [115], cannabidiols [116], and of course of several peptides [48,110,117,118,119].

The most important feature of the MCSGP process is the possibility to use a solvent gradient for the elution, differently than other continuous chromatographic techniques that only work in isocratic conditions, for example Simulated Moving Bed [25,120]. This requirement is mandatory because the retention of biomolecules strictly depends on the mobile phase composition (percentage of organic modifier, salt concentration, etc.), as already stated above [49,105,118,121,122,123,124].

**Figure 7:**
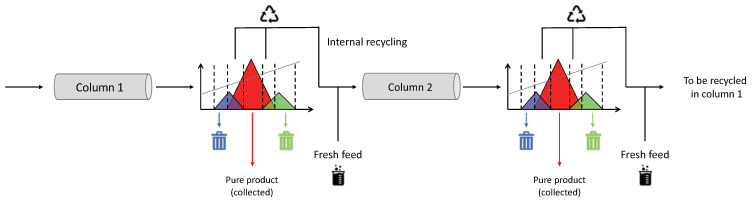
A scheme illustrating the MCSGP technique is shown. The gradient elution is performed in the first column. The main peak (red) overlaps in the front and in the tail with other groups of impurities (blue and green). The overlapping regions are therefore internally recycled into the second columns in order not to waste product, whereas the central part of the red peak (pure target product) is collected. On the contrary, windows where only impurities elute with no product are discarded. The same procedure is repeated at the outlet of the second column, where the overlapping regions are recycled into the first one. During the recycling, also some fresh feed is loaded into the same column, in order to keep the total amount of product constant into the system.

The improvements gained with MCSGP are due to the fact that the overlapping regions (the regions where product partially elutes with weak and strong impurities, on the head and on the tail of the main peak respectively) are recycled internally into the unit in order to be reprocessed. The mechanism regulating the internal recycling lies in the movement (switching) of the inlet and outlet column valves, which can connect and disconnect the columns and, as a consequence, change the path executed by the mobile phase. In Figure 7, a scheme illustrating the working principle of MCSGP is depicted.

**Figure 8:**
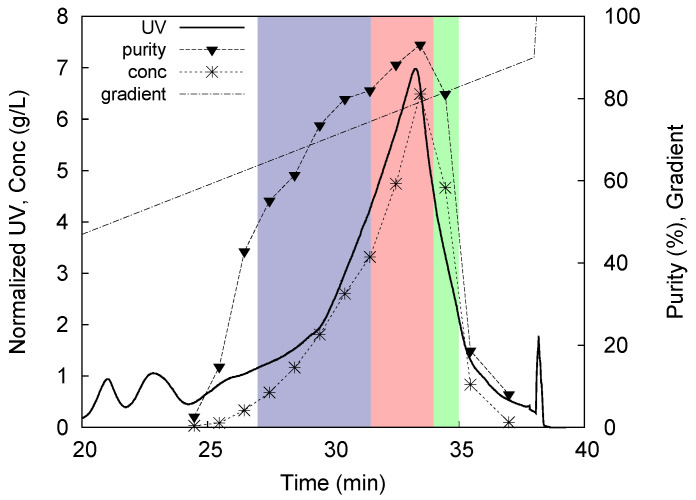
Batch chromatogram obtained for the purification of a glucagon crude mixture. The blue and green windows represent the impure portions of the main peak that need to be recycled into the second column. On the other side, the red window represents the target product fulfilling the purity requirements. Beside the UV profile, also the purity and concentration profiles and the gradient are shown. For interpretation of the references to colors in this figure legend, the reader is referred to the web version of this article. Reproduced with permission from [48].

The combination of continuous and countercurrent concepts coupled with the internal recycling and the possibility to use a solvent gradient program enables separation of ternary mixtures and allows to reach high product purities and elevated yields at the same time, outperforming the traditional single column processes, in many cases also in terms of productivity [125,126]. One of the first applications of the six-column MCSGP process concerned the purification of a 32 amino acids synthetic peptide, namely calcitonin, whose initial purity was 46%. It was found that, using a reversed-phase method, the yield increased from 66% for the batch purification to almost 100% for the continuous process, with a purity of 93% [110]. Another case of a complex peptide mixture that has been successfully separated through MCSGP is an industrial crude of glucagon, a synthetic peptide containing 29 residues. The study published [48] shows how to transfer a batch method to an MCSGP process; Figure 8 illustrates the batch chromatogram starting from which the MCSGP method has been setted. The results obtained indicate that the recovery can be increased from 71% to almost 88% with twin-column MCSGP, reaching a purity value equal to 89%. Last, a very recent research [119] reports an application where MCSGP has led to outstanding results in all the performance parameters. In the case of purification of icatibant in reversed-phase conditions, indeed, the purity obtained for the target product was greater than 99% for both the batch and the MCSGP method, but the continuous processes allowed to improve the recovery from around 12% to more than 95%, with a process gain of +670% in terms of recovery. Additionally, the productivity was improved by more than 5 times whereas the solvent consumption was reduced by 80%. For an in-depth description of the MCSGP technique, further examples of application and how to develop an MCSGP method starting from a design batch chromatogram, the interested reader is referred to a review specifically focused on this topic, written by some of the authors [24].

## 5. Conclusions

Bioactive peptides show a broad range of activities, that vary from antimicrobial, antihypertensive, immunomodulatory, antioxidant, etc; therefore, they can be employed as food additives or pharmaceutical ingredients for the therapy of some diseases. They can be produced by means of hydrolysis of the parent protein or they can be chemically synthesized, especially through Solid Phase Peptide Synthesis. Their application as biopharmaceuticals requires the peptide to fulfill very strict purity and quality specifications; as a consequence, choosing proper purification processes is one of the most important parts of the manufacturing process design. Liquid chromatography is usually the best technique employed for purification, especially when used in reversed-phase mode. Additionally, ion-exchange chromatography and hydrophilic interaction chromatography are frequently used, depending on the hydrophobic/hydrophilic properties of the peptide and on its charge. Innovative mixed-mode stationary phases, on the other hand, combine reversed-phase and ion-exchange features. So, depending on the chemical characteristics of the peptide, for a large class of compounds a suitable chromatographic technique can be easily found.

Thanks to important technological advancements, continuous chromatographic techniques based on the concept of countercurrent chromatography are becoming established. MCSGP represents a remarkable case of process which is sparking interest, because of improvements gained in product quality, economic advantages related to higher productivity and, nonetheless, a simplification of the process due to the automation of operations.

Although huge progress has been done in the purification processing, theoretical studies focusing on the modeling of the process should be implemented, to favour a quick optimization of the procedures. Additionally, some obstacles and barriers must still be faced from the point of view of the regulation, in order to assure to meet Good Manufacturing Practise (GMP). 

## Figures and Tables

**Table 1:** In this table, the main techniques employed for peptide purification and their interaction mechanisms are summarized.

Purification or	Mechanism
Identification Method	
Ultrafiltration	Target peptide is separated from other species depending on their size
IsoElectroFocusing (IEF)	Peptides are separated on the basis of their isoelectric point through an electric field and a pH gradient
Single-column chromatography	Different modes of chromatography have been developed, depending on the chemical features of the analytes: RP-LC: hydrophobic character Ion-Exchange: charge HILIC: hydrophilic character Mixed-Mode: combination of two ligands on the same stationary phase with orthogonal interection mechanisms
Multicolumn chromatography	Combination of two or more orthogonal chromatographic modes applied consecutively
MCSGP	Same separation principles as single-column chromatography but with the use of two or more identical columns. The performance parameters increase due to internal recycling of impure fractions into the system.

## Data Availability

Not applicable.
